# Supplementary material for: Development of a Versatile, Near Full Genome Amplification and Sequencing Approach for a Broad Variety of HIV-1 Group M Variants
Source: Viruses. 2019 Apr 1;11(4):317. doi: 10.3390/v11040317 (PMC6520859; doi:10.3390/v11040317)
Supplement: Supplementary file 1 [file viruses-11-00317-s001.pdf]

Supplementary Figures

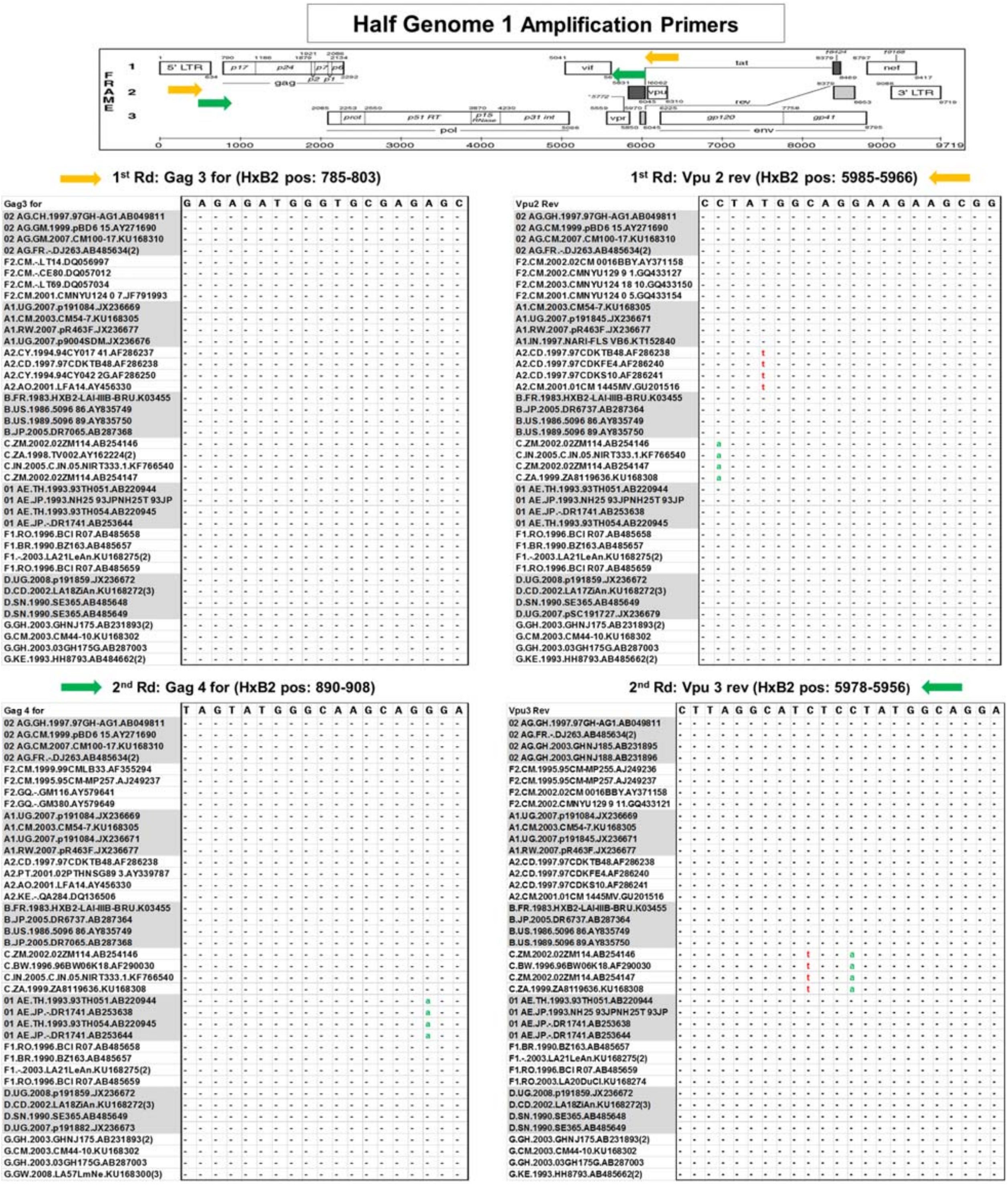

Supplementary Figure S1. Conserved primer binding sites for HIV-1 half genome 1 amplification. Multiple sequence alignments of primer binding sites across ten different pure subtypes and CRFs, as relevant for the half genome 1 (HG1) amplification approach HG1b. For each subtype/CRF, four reference sequences were selected that broadly cover the respective clade. The numbering of primer binding regions is based on the HxB2 reference strain [GenBank: K03455]. Positions (pos) of primer binding sites are shown in brackets (HxB2 pos. 908- 5956). Orange and green arrows along the HxB2 genome map indicate first round and second round primer positions, respectively. Black dots and colored letters indicate matches and mismatches with primer nucleotides shown on top, respectively.

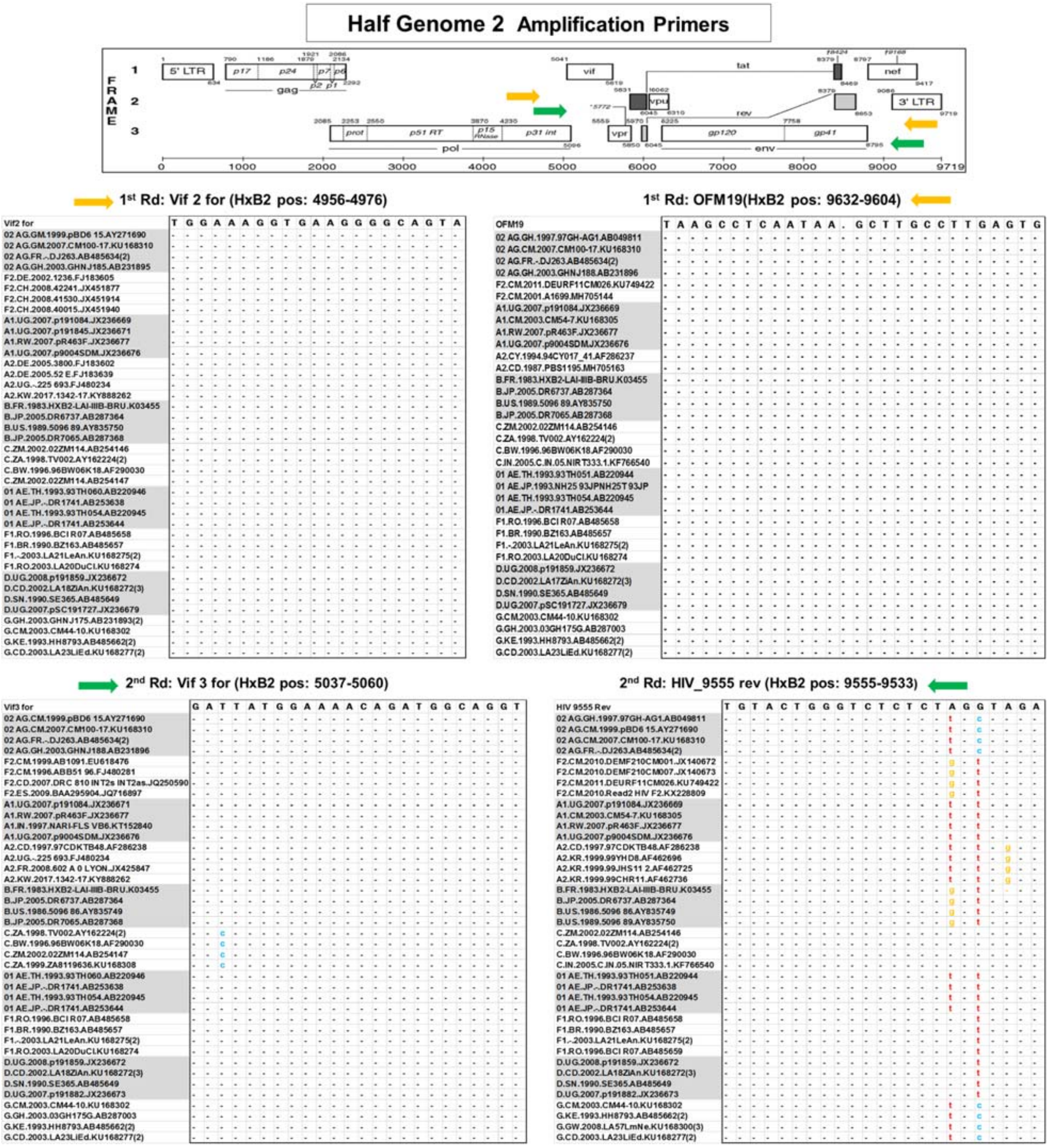

Supplementary Figure S2. Conserved primer binding sites for HIV-1 half genome 2 amplification. Multiple sequence alignments of primer binding sites across ten different pure subtypes and CRFs, as relevant for the half genome 2 (HG2) amplification approach. For each subtype/CRF, four reference sequences were selected that broadly cover the respective clade; for the binding site of primer 3'UTR rev 1, only two A2 and F2 reference sequences were available from the

LANL database. The numbering of primer binding regions is based on the HxB2 reference strain [GenBank: K03455]. Positions (pos) of primer binding sites are shown in brackets (HxB2 pos. 5060-9533). Orange and green arrows along the HxB2 genome map indicate first round and second round primer positions, respectively. Black dots and colored letters indicate matches and mismatches with primer nucleotides shown on top, respectively.

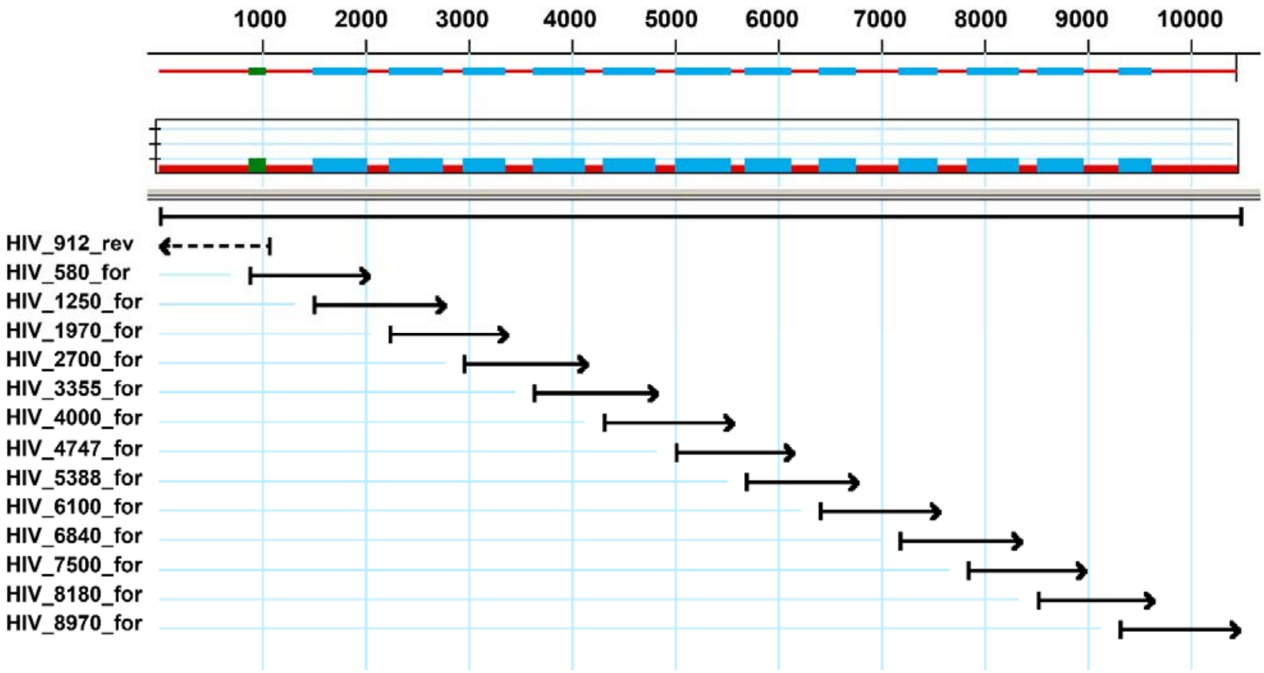

Supplementary Figure S3. Stepwise sequencing approach: Strategy view from SeqMan Pro (DNAstar package) illustrating the sequencing process for near full genomes using 14 frequently applied sequencing primers (13 forward primers and one reverse primer). Primers were named according to the location of their first 5′ nucleotide in the HxB2 reference strain [GenBank: K03455]. Black arrows indicate read lengths. Red bars on top of the diagram indicate sequence coverage by only one read. Blue and green bars indicate an overlap between two reads.

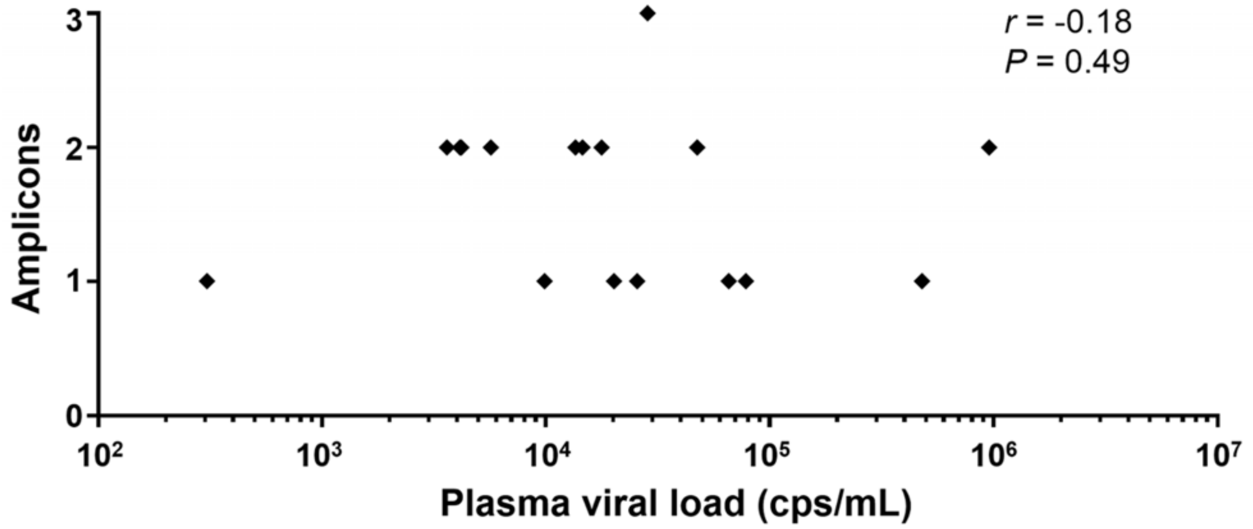

Supplementary Figure S4. Correlation analysis between plasma viral load and the number of amplicons needed for near full genome sequencing. Dot plot analysis displaying viral loads from URF plasma samples on the x-axis plotted against the number of required amplicons for near full genome sequencing on the y-axis (see also Table 1 in Banin et al., JIAS 2019 b). Correlation coefficients *r* and *P*-values were calculated using a two-tailed, non-parametric Spearman rank test.
